# The multiple alignments of very short sequences

**DOI:** 10.1096/fba.2020-00118

**Published:** 2021-04-29

**Authors:** Kristóf Takács, Vince Grolmusz

**Affiliations:** ^1^ PIT Bioinformatics Group Eötvös University Budapest Hungary; ^2^ Uratim Ltd Budapest Hungary

## Abstract

The multiple sequence alignment (MSA) is an increasingly important task in bioinformatics as we have to deal with the constantly increasing gene‐ and protein sequence databases. MSA is applied in phylogenetic analysis, in discovering conservative protein domains, in the assignment of secondary and tertiary structural features in proteins, or in the metagenomic sample analysis and gene discovery. Usually, the focus is on the MSA of long sequences, since in the practice these tasks appear most frequently. However, the strict analysis of the optimal MSA of short sequences is an area of negligence, and findings there may contribute to better and faster algorithms for the multiple alignment of long sequences. In the present contribution, we are examining length‐1 sequences using arbitrary metric and length‐2 sequences using unit metric, and we show that the optimum of the MSA problem can be achieved by the trivial alignment in both cases.

## 1 INTRODUCTION

Multiple sequence alignment (MSA) is one of the central problems in classical bioinformatics. While the exact optimal global and local alignments of two sequences can be computed in quadratic time with the Neddleman‐Wunsch^1^ and the Smith‐Waterman^2^, ^3^ algorithms, respectively, the exact multiple alignment generally is proven to be an NP‐hard problem,^4^ and therefore, it is very unlikely to be computable in $n^{\alpha }$‐time algorithm, for any constant α, where n denotes the input size.

Heuristic MSA algorithms include the different versions of CLUSTAL,^5^, ^6^, ^7^, ^8^, ^9^, ^10^, ^11^ MSACompro,^12^ PRALINE,^13^ TCS,^14^ PASTASpark,^15^ and numerous others.

Multiple sequence alignment algorithms have a range of applications in bioinformatics, for example, in HMM profile building in the famous HMMER suite of programs,^16^, ^17^, ^18^, ^19^ in identifying conservative protein‐ and genome‐sequences, in phylogenetic tree building and analysis,^20^, ^21^, ^22^, ^23^ motif discovery and gene identification in metagenomic samples,^19^ secondary protein structure prediction,^24^, ^25^ and solvent accessibility computation.^26^

In general, the MSA problem is computationally hard (i.e., NP‐hard).^4^ An interesting question is when does the problem become a hard instance when the parameters are modified?

It is known that for a constant number of sequences, the MSA problem is solvable in polynomial time by dynamic programing algorithms (Needleman‐Wunsch and Smith‐Waterman generalizations). Therefore, the MSA problem is “easy”, that is, polynomial time computable, if the number of the sequences is small (i.e., it is a constant). To the best of our knowledge, no one examined the complexity of MSA when the number of the sequences is not small, but their length is.

One can assume that for length‐1 (and perhaps even for length‐2) sequences, it may not be that hard to find an optimal alignment. Furthermore, if an optimal alignment for short sequences can be determined in polynomial time, then it could also help to develop faster or more accurate heuristic algorithms. In this work, some new results regarding the alignment of short sequences are presented.

### 1.1 Definitions and notations

Definition 1 *Let* $\Sigma =\left\{a_{1},\ldots ,a_{n}\right\}$ *be a finite alphabet; a string over* Σ *is called a sequence. The pair of sequences* $s_{1}^{\prime },s_{2}^{\prime }$ *is an alignment of sequences* $s_{1}$ *and* $s_{2}$ *if for* $i=1,2:s_{i}^{\prime }$ *is obtained from* $s_{i}$ *by inserting gaps (spaces, denoted by –) into or at either end of* $s_{i}$ *and after that,* $s_{1}^{\prime }$ *and* $s_{2}^{\prime }$ *have the same length. It is assumed that "—" is not an element of alphabet* Σ.

The alignment of Definition 1 consists of two sequences of the same length. Consequently, every character of $s_{1}^{\prime }$ is uniquely corresponded to a character of $s_{2}^{\prime }$, simply by locating at the same position.

Let ℓ be the common length of $s_{1}^{\prime }$ and $s_{2}^{\prime }$. The *cost* of this alignment is

(1) $$\text{cost}\left(s_{1}^{\prime },s_{2}^{\prime }\right)=\sum_{i=1}^{\ell }d\left(s_{1}^{\prime }\left(i\right),s_{2}^{\prime }\left(i\right)\right),$$

where *d* is a *score scheme* over $\Sigma \cup \left\{‐\right\}$, and $s_{j}^{\prime }\left(i\right)$ is the *i*th character of $s_{j}^{\prime }$. The score scheme is usually required to be a metric on the set $\Sigma \cup \left\{‐\right\}$, that is, it needs to satisfy $d\left(\left(u,v\right)\right)=0\Leftrightarrow u=v;\;d\;\left(\left(u,v\right)\right)=\;d\;\left(\left(v,u\right)\right)$; and the triangle inequality: $d\left(u,w\right)\leq \;d\;\left(u,v\right)+\;d\;\left(v,w\right)$, $\forall u,v,w\in \Sigma \cup \left\{‐\right\}$. A frequently used score scheme is the *unit metric*, where $\;d\;\left(u,v\right)=0$ if u=v and 1 otherwise. We call an alignment *optimal* for two sequences if its cost is minimal among every possible alignments.

The definition of aligning two sequences can easily be generalized for more strings: let k≥2 be a positive integer, and suppose that we want to align the sequences $s_{1},s_{2},\ldots ,s_{k}$. Let us insert gaps into or at either end of strings $s_{1},s_{2},\ldots ,s_{k}$, so that they have the same length ℓ, and in the proper order, write the k sequences $s_{1}^{\prime },s_{2}^{\prime },\ldots ,s_{k}^{\prime }$, each of length ℓ, under one another. This table can be considered a matrix of size k×ℓ, and it is called a *multiple alignment* of sequences $s_{1},s_{2},\ldots ,s_{k}$. Different *scoring methods* can be applied for multiple alignments, perhaps the most often used one is the *sum of pairs* method, where the cost is the sum of the costs of the alignments of the $\left(\begin{array}{c} k\\ 2 \end{array}\right)$ pairs from the aligned sequences. More exactly, if $s_{1},\ldots ,s_{k}$ are sequences to be aligned, then their sum of pair cost^27^ is

(2) $$\sum_{i=1}^{k‐1}\sum_{j=i+1}^{k}\text{cost}\left(s_{i}^{\prime },s_{j}^{\prime }\right).$$

*Examples*. (i) Let $S\;:=\left\{CCG,GCG,CGC\right\}$. The following set of sequences is a multiple alignment A of S:

**Table:** 

C	C	G	–
G	C	G	–
–	C	G	C

Using the unit metric and computing the costs of the columns, cost(A)=3+0+0+2=5.

(ii) Let Σ now contain only two characters (C and G) with the following metric:

**Table:** 

	C	G	–
C	0	2	1
G	2	0	1
–	1	1	0

Let $S:=\left\{CG,GC,GG\right\}$. A multiple alignment A of S:

A =

**Table:** 

–	C	G
G	C	–
G	–	G

Using the given metric, cost $\left(A\right)$ is equal to 2+2+2=6.

## 2 MULTIPLE SEQUENCE ALIGNMENT FOR LENGTH‐1 SEQUENCES

In this section, we focus on aligning length‐1 sequences (equivalently, characters of Σ). An important earlier result needs to be quoted here^28^:

Theorem 2 (Lemma 3) Let U be a subset of a set S of sequences over Σ, such that U contains only identical sequences, and let A be an optimal alignment of S. Let $A_{U}$ denote the restriction of A to the rows of U. Then

$$\cos t\left(A_{U}\right)=\sum_{u_{i}\in U}\sum_{\begin{array}{c} u_{j}\in U\\ i<j \end{array}}d\left(u_{i},u_{j}\right)=0.$$

An important corollary of this theorem is the following one: it is enough to examine the sets of pairwise different sequences because in each optimal alignment, every instance of a given sequence is aligned identically.

The next definition will be used frequently throughout this work:

Definition 3 *Let S be a set of sequences that have the same length.* A *is called the trivial alignment of S if* A *is constructed by writing every sequence under each another, without using any gaps*.

### 2.1 Multiple sequence alignment for length‐1 sequences using unit metric

The main result of this subsection is the next theorem:

Theorem 4 Using unit metric, there cannot be a multiple sequence alignment for length‐1 sequences that has cost less than the cost of their trivial alignment. Additionally, if we align k pairwise different length‐1 sequences, then the cost of an optimal alignment is $\begin{array}{c} \left(\begin{array}{c} k\\ 2 \end{array}\right) \end{array}$.

By Theorem 2, we may assume that the characters to be aligned are pairwise different. It is easy to see that the trivial alignment of k different characters has a cost of $\left(\begin{array}{c} k\\ 2 \end{array}\right)$: there are $\left(\begin{array}{c} k\\ 2 \end{array}\right)$ pairs among these characters and in every pair, there are two different sequences, so the cost of an aligned pair is always 1.

Let us suppose that this alignment is not optimal, then the length of every aligned sequence must be at least 2 in an optimal alignment. If this common length of aligned sequences is ℓ≥2, then the general structure of the n×ℓ matrix of this multiple alignment is as follows: ∀i:1≤i≤ℓ, there are $k_{i}$ characters in the *i*th column, where $\sum_{i=1}^{\ell }k_{i}=k$, and they are placed so that in each row, there is only one character and ℓ‐1 gaps (see Table 1).

**TABLE 1:** A multiple alignment for length‐1 sequences on ℓ columns

$a_{1}$	–	…	–
…	…	…	…
$a_{k_{1}}$	–	–	–
–	$a_{k_{1}+1}$	…	–
…	…	…	…
–	$a_{k_{2}}$	…	–
…	…	…	…
–	–	…	$a_{k_{\ell ‐1}+1}$
…	…	…	…
–	–	…	$a_{k_{\ell }}$

Obviously, the cost of the first column is

$$\left(\begin{array}{c} k_{1}\\ 2 \end{array}\right)+\left(k‐k_{1}\right)k_{1}$$

since there are *k*_1_ different characters with cost of $\left(\begin{array}{c} k_{1}\\ 2 \end{array}\right)$, and besides that, all of the $\left(k‐k_{1}\right)$ gaps increase the cost by one with every alphabetical character. A similar statement is true for every column, so the cost of this alignment is:

$$\sum_{i=1}^{\ell }\left(\begin{array}{c} k_{i}\\ 2 \end{array}\right)+\left(k‐k_{i}\right)k_{i}=k\sum_{i=1}^{\ell }k_{i}‐\frac{1}{2}\left(\sum_{i=1}^{\ell }k_{i}+\sum_{i=1}^{\ell }k_{i}^{2}\right)=k^{2}‐\frac{1}{2}\left(k+\sum_{i=1}^{\ell }k_{i}^{2}\right)$$

Consequently, the cost above is minimized, when $\sum_{i=1}^{\ell }k_{i}^{2}$ is maximized. Since

$$k^{2}={\left(\sum_{i=1}^{\ell }k_{i}\right)}^{2}=\sum_{i=1}^{\ell }k_{i}^{2}+2\sum_{i=1}^{\ell }\sum_{\begin{array}{c} j=1\\ i<j \end{array}}^{\ell }k_{i}k_{j},$$

holds, it is clear that $\sum_{i=1}^{\ell }k_{i}^{2}=k^{2}$, and the cost of this alignment cannot be less than $\left(k^{2}‐k\right)/2=\left(\begin{array}{c} k\\ 2 \end{array}\right)$, that is, the cost of the trivial alignment.

Note: From the proof, it is also clear (by minimizing $\sum_{i=1}^{\ell }k_{i}^{2}$) that a multiple alignment for k different length‐1 sequences cannot have a higher cost than $k^{2}‐k/2‐k^{2}/\left(2\ell \right)$, if the length of aligned sequences is ℓ. Since ℓ≤k, the cost can be at most $k^{2}‐k$ and this limit can be reached if there is only one character in every column and in every row, then the cost is $k\left(k‐1\right)=k^{2}‐k$.

## 3 MULTIPLE SEQUENCE ALIGNMENT FOR LENGTH‐1 SEQUENCES USING ARBITRARY METRIC

In this subsection, it will be shown that for length‐1 sequences, we can use any metric as a score scheme, and the MSA problem still remains as easy as in the case of the unit metric.

Theorem 5 Using arbitrary metric, the minimum cost of the multiple sequence alignment for length‐1 sequences is attained by the trivial alignment, and if k different sequences are aligned, *then the optimal* cost is equal to.

$$C=\sum_{i=1}^{k}\sum_{\begin{array}{c} j=2\\ i<j \end{array}}^{k}d(a_{i},a_{j}).$$

Because of Theorem 2, it can be assumed again that every sequence has exactly one instance in the set *S* of sequences to be aligned. If we consider the trivial alignment of the *S*, it is easy to see that its cost is equal to *C*. Induction for the number of the columns in a MSA will be used to show that no alignment can have lower cost than *C*.

Let be assumed that the trivial alignment is not optimal, and let A denote an optimal alignment. If A is not the trivial alignment, then A has ℓ columns where ℓ≥2. It can be shown that A cannot have exactly two columns, because in this case, the trivial alignment would have a lower cost than A has.

Let us assume to the contrary that A has exactly two columns; so there are $k_{1}$ sequences in the first column and $k_{2}$ in the second column, where $k_{1}+k_{2}=k$ and there is exactly one character in each row (since our sequences to be aligned have length equal to 1, see Table 2).

**TABLE 2:** A multiple alignment for *k* length‐1 sequences in two columns

$a_{1}$	–
$a_{2}$	–
—	…
$a_{k_{1}}$	–
–	$a_{k_{1}+1}$
–	$a_{k_{1}+2}$
…	…
–	$a_{k}$

We assume, without loss of generality, that the sequences in the first column are $a_{1},a_{2},\ldots ,a_{k_{1}}$ and every other sequences are placed in the second column. If the cost of the first column of A is denoted by $\text{cost}\left(1\right)$, then

$$\text{cost}\left(1\right)=\sum_{i=1}^{k_{1}}\sum_{\begin{array}{c} j=2\\ i<j \end{array}}^{k_{1}}d(a_{i},a_{j})+k_{2}\sum_{i=1}^{k_{1}}d\left(a_{i},-\right).$$

Similarly, the cost of the second column is

$$\text{cost}\left(2\right)=\sum_{i=k_{1}+1}^{k}\sum_{\begin{array}{c} j=k_{1}+2\\ i<j \end{array}}^{k}d(a_{i},a_{j})+k_{1}\sum_{j=k_{1}+1}^{k}d\left(a_{j},-\right).$$

and cost(A)=cost(1)+cost(2).

A lower bound for $\text{cost}\;\left(A\right)$ can be determined by pairing the $d\left(a_{i},‐\right)$ summands in $\;\text{cost}\left(1\right)$ to the summands of same form in $\text{cost}\left(2\right)$ and using the triangle inequality. For example, for a fix *i* ($1\leq i\leq k_{1}$) and $\forall j:k_{1}+1\leq j\leq k$, it is true that $d\left(a_{i},‐\right)+\;d\left(a_{j},‐\right)\geq \;d\left(a_{i},a_{j}\right)$, so

$$k_{2}d\left(a_{i},‐\right)+\sum_{j=k_{1}+1}^{k}d(a_{j},‐)\geq \sum_{j=k_{1}+1}^{k}d(a_{i},a_{j}).$$

It is useful to notice that the summands on the right side of this inequality are exactly those ones that are not included in $\;\text{cost}\left(1\right)$ when we consider summands of the form of $d\left(a_{i},a_{j}\right)$ for this fix *i*.

By considering this inequality for every $1\leq i\leq k_{1}$, the following lower bound can be given:

$$k_{2}\sum_{i=1}^{k_{1}}d(a_{i},‐)+k_{1}\sum_{j=k_{1}+1}^{k}d(a_{j},‐)\geq \sum_{i=1}^{k_{1}}\sum_{j=k_{1}+1}^{k}d(a_{i},a_{j}),$$

This implies that

$$\cos t\left(A\right)\geq \sum_{i=1}^{k_{1}}\sum_{\begin{array}{c} j=2\\ i<j \end{array}}^{k_{1}}d\left(a_{i},a_{j}\right)+\sum_{i=k_{1}+1}^{k}\sum_{\begin{array}{c} j=k_{1}+2\\ i<j \end{array}}^{k}d\left(a_{i},a_{j}\right)+\sum_{i=1}^{k_{1}}\sum_{j=k_{1}+1}^{k}d\left(a_{i},a_{j}\right)=\sum_{i=1}^{k}\sum_{\begin{array}{c} j=2\\ i<j \end{array}}^{k}d\left(a_{i},a_{j}\right)=C.$$

It is assumed that the trivial alignment with cost C is not optimal; therefore, A cannot be an optimal alignment of S. By this contradiction, it is proved that an optimal alignment of *S* cannot have exactly 2 columns.

Using induction, we assume that it is shown ∀i:2≤i<ℓ that an optimal alignment cannot have exactly *i* columns, and let A be an optimal alignment with ℓ columns. Considering the cost of the first two columns of A, there are $k_{1}$ sequences in the first column and $k_{2}$ sequences in the second one. It is enough to prove that by merging these two columns, the cost of the new alignment is lower than the cost of A. The cost of these columns (see Table 3) in A is equal to

$$\sum_{i=1}^{k_{1}}\sum_{\begin{array}{c} j=2\\ i<j \end{array}}^{k_{1}}d\left(a_{i},a_{j}\right)+\left(k‐k_{1}\right)\sum_{i=1}^{k_{1}}d\left(a_{i},‐\right)+\sum_{i=k_{1}+1}^{k_{2}}\sum_{\begin{array}{c} j=k_{1}+1\\ i<j \end{array}}^{k_{2}}d\left(a_{i},a_{j}\right)+\left(k‐k_{2}\right)\sum_{i=k_{1}+1}^{k_{2}}d\left(a_{i},‐\right).$$

**TABLE 3:** The first two columns of A

$a_{1}$	–
$a_{2}$	–
…	…
$a_{k_{1}}$	–
–	$a_{k_{1}+1}$
–	$a_{k_{1}+2}$
…	…
–	$a_{k_{1}+k_{2}}$
–	–
…	…
–	–

Let us focus on the first $k^{\prime }=k_{1}+k_{2}$ characters of these columns. It is an alignment of $\left\{a_{1},a_{2},\ldots ,a_{k\prime }\right\}$ on two columns and it was shown that if these sequences are aligned trivially instead of using two columns, then the cost of the alignment cannot be higher. It means the following:

$$\begin{array}{c} \sum_{i=1}^{k_{1}}\sum_{\begin{array}{c} j=2\\ i<j \end{array}}^{k_{1}}d\left(a_{i},a_{j}\right)+k_{2}\sum_{i=1}^{k_{1}}d\left(a_{i},‐\right)+\sum_{i=k_{1}+1}^{k^{\prime }}\sum_{\begin{array}{c} j=k_{1}+1\\ i<j \end{array}}^{k^{\prime }}d\left(a_{i},a_{j}\right)\\ \;+k_{1}\sum_{i=k_{1}+1}^{k^{\prime }}d\left(a_{i},‐\right)+\left(k‐k\right)\sum_{i=1}^{k_{1}}d\left(a_{i},‐\right)\\ \;+\left(k‐k^{\prime }\right)\sum_{i=k_{1}+1}^{k^{\prime }}d\left(a_{i},‐\right)\geq \sum_{i=1}^{k^{\prime }}\sum_{\begin{array}{c} j=2\\ i<j \end{array}}^{k^{\prime }}d\left(a_{i},a_{j}\right)+\left(k‐k^{\prime }\right)\sum_{i=1}^{k^{\prime }}d\left(a_{i},‐\right) \end{array}$$

On the left side of this inequality, there is the cost of the first two columns of A, while on the right side, there is the cost of the column that is constructed by merging the first two columns of A. Therefore, a lower bound for $cost\left(A\right)$ is given by an alignment that has l‐1 columns, implying that A is not optimal W.

## 4 MULTIPLE SEQUENCE ALIGNMENT FOR LENGTH‐2 SEQUENCES

In this section, it will be shown that using the unit metric, a set of length‐2 sequences cannot be aligned with less cost than their trivial alignment; however, this statement does not hold for using arbitrary metric.

Theorem 6 Using the unit metric, no multiple sequence alignment for length‐2 sequences has less cost than their trivial alignment. If we align k different sequences $\left(s_{1}=a_{i_{1}}a_{i_{k+1}},s_{2}=a_{i_{2}}a_{i_{k+2}},\ldots ,s_{k}=a_{i_{k}}a_{i_{2k}}\right)$, then the cost of the optimal alignment is.

$$\sum_{j=1}^{k}\sum_{\begin{array}{c} \ell =2\\ j<\ell \end{array}}^{k}d\left(a_{i_{j}},a_{i_{\ell }}\right)+\sum_{j=k+1}^{2k}\sum_{\begin{array}{c} \ell =k+2\\ j<\ell \end{array}}^{2k}d\left(a_{i_{j}},a_{i_{\ell }}\right).$$

Let S denote the set of sequences that need to be aligned. It is clear that the trivial alignment of S has the cost written above, so this lower bound is accessible. In other words, it is enough to prove that for any S, a non‐trivial alignment cannot have less cost than the trivial one.

Let A be an alignment of S on ℓ columns where ℓ≥3. Let the rows of A be permuted, so that those aligned sequences, where the indices of the two non‐gap characters are the same, are placed under each other, forming a block of sequences. This operation does not change the cost of A. In every row of A, there are exactly two characters and ℓ‐2 gaps, so there can be $\left(\begin{array}{c} \ell \\ 2 \end{array}\right)$ types of aligned sequences in A, considering only the positions of the non‐gap characters in a row. This implies that there will be $\left(\begin{array}{c} \ell \\ 2 \end{array}\right)$ (not necessarily non‐empty) blocks after permuting the rows of A (e.g., if ℓ=4, then there are $\left(\begin{array}{c} 4\\ 2 \end{array}\right)=6$ blocks after the permutation of the rows, see Table 4).

**TABLE 4:** The structure of A after permuting its rows and making its block setting with ℓ=4. Number 1 denotes the first characters, and number 2 the second letters. During the proof, an upper bound is given for the cost of aligning letters with the same order that are not aligned in A by using character‐gap alignment costs that are included in cost $\left(A\right)$

1	2	–	–
…	…	…	…
1	2	–	–
1	–	2	–
…	…	…	…
1	–	2	–
1	–	–	2
…	…	…	…
1	–	–	2
–	1	2	–
…	…	…	…
–	1	2	–
–	1	–	2
…	…	…	…
–	1	–	2
–	–	1	2
…	…	…	…
–	–	1	2

After making this block setting, it is clear that there are six types of aligned character pairs in A:

1. first characters of some sequences aligned with other sequences’ first characters;
2. first characters of some sequences aligned with other sequences’ second characters;
3. first characters of some sequences aligned with gaps;
4. second characters of some sequences aligned with other sequences’ second characters;
5. second characters of some sequences aligned with gaps;
6. gaps aligned with gaps.

In the trivial alignment T, there are only pairs of types (i) and (iv); moreover, *every* sequence's first character is aligned with each another in T (and it holds similarly for *every* second character of the sequences of S). Nevertheless, in a non‐trivial alignment A, there are aligned sequences whose first or second characters are not aligned with each other in A. This implies that it is enough to give an upper bound for the cost of these characters in T that are aligned with each other in T but are not aligned with each other in A, using parts of cost(A) for this bound. (Because every part of cost (A) is non‐negative, if a bijection can be given between the letter‐letter alignments in T that are not aligned in A and some other alignments of characters of A (not excluded character‐gap alignments), so that the latter alignments have always at least as much cost as the former ones, then it means that cost $\left(A\right)\geq$ cost(T).)

If d denotes the unit metric, then the following inequality holds for every pair of sets P,R on arbitrary alphabet (where P and R can contain a letter more than once):

$$\sum_{a_{i_{j}}\in P}\sum_{a_{i_{l}}\in R}d\left(a_{i_{j}},a_{i_{l}}\right)\leq \left|P\right|\sum_{a_{i_{l}}\in R}d\left(a_{i_{l}},‐\right)=\left|P\right|\left|R\right|.$$

Using this inequality, a bijection mentioned above can be given: first, let be considered two sequences whose first characters ($a_{i}$ and $a_{j})$ are not aligned in A (it can be assumed that $a_{j}$ has bigger column index). This implies that the element that is in the intersection of the row of $a_{j}$ and the column of $a_{i}$ must be a gap. $d\left(a_{i},a_{j}\right)\leq d\left(a_{i},‐\right)$, so the cost of the alignment of $a_{i}$ and $a_{j}$ in T can be estimated by the cost of the alignment of two characters in A.

Similarly, if two sequences are considered whose second characters $(a_{i}$ and $a_{j})$ are not aligned in A, then (assuming that $a_{j}$ has bigger column index) the element in the intersection of the row of $a_{i}$ and the column of $a_{j}$ must be a gap. The same estimation can be given like before, meaning that the cost of the alignment of $a_{i}$ and $a_{j}$ in T is less or equal to the cost of a character‐gap alignment in A.

Considering the block setting (Table 4) of A, let $B_{i}$ and $B_{j}$ be the two blocks whose sequences’ first characters are not aligned in A. Assuming that the first characters of sequences in $B_{j}$ have bigger column index, there must be $\left|B_{j}\right|$ gaps in the intersection of the column of the first characters of sequences in $B_{i}$ and the rows of $B_{j}$. If we denote the first letters of the sequences of $B_{i}\left(B_{j}\right)$ by $a_{b_{i}}\left(a_{b_{j}}\right)$, then (because of the statements of the latter two paragraphs) the following holds:

$$\sum_{b_{i}\in B_{i}}\sum_{b_{j}\in B_{j}}d\left(a_{b_{i}},a_{b_{j}}\right)\leq \left|B_{j}\right|\sum_{b_{i}\in B_{i}}d\left(a_{b_{i}},‐\right)=\left|B_{i}\right|\left|B_{j}\right|$$

Besides that, a similar result can be established if we consider two blocks whose sequences’ second characters are not aligned, using the gaps of the block that has the column with smaller column index (see Table 5). By these estimations, it is clear that this assignment between the character–character alignments in T, which are not present in A, and character‐gap alignments in A lead to a result that the latter costs in A cannot be less than the corresponding costs in T. We also need to show that this assignment is a bijection, that is, there are no character‐gap alignments that are used more than one time.

**TABLE 5:** The block setting of A if ℓ=4, denoting only that an element is the first/second character of its aligned sequence or a gap. For example, the first element of the first row in the block setting and the second element of the fourth row (which are denoting the first characters of some sequences) are not aligned in A, so the cost of their alignment with each other, which is a part of cost $\left(T\right)$ but not a part of cost $\left(A\right)$, must be estimated from above with a part of cost $\left(A\right)$. Namely, with the cost of aligning the block setting's first element of the first row with the gaps in the first element of the fourth row

1	2	–	–
1	–	2	–
1	–	–	2
–	1	2	–
–	1	–	2
–	–	1	2

A set of gaps in the block setting are considered in an estimation if and only if some characters in the block that are containing these gaps and some characters from another block that are aligned in the same column must be aligned in T but they are not aligned in A. This implies that these gaps are not used in estimations like above more times than the alignment of this gap set with the rest of the given column. Therefore, the former assignment is a bijection, implying that cost $\left(A\right)\geq$ cost $\left(T\right)$.W.

In the proof, only the following property of the unit metric has been used: $\forall a_{i},a_{j}\in \Sigma :d\left(a_{i},a_{j}\right)\leq d\left(a_{i},‐\right)$. It follows that Theorem 6 remains valid for any metric, satisfying this property.

As the next example shows, the trivial alignment will not always be optimal for length‐2 sequences if an arbitrary metric is used. Let Σ contain two characters (C and G) with the same metric on Σ as in the Example $\left(\mathrm{ii}\right)$ at the end of the Introduction. Let S be also the same as in Example $\left(\mathrm{ii}\right)$: $S=\left\{CG,GC,GG\right\}$. The trivial alignment of S has a cost of 8, but as Table 6 shows, there is an alignment of S that has cost only of 6.

**TABLE 6:** The trivial and an optimal alignment of S

C	G	–	C	G
G	C	G	C	–
G	G	G	–	G

In the previous section, it was shown that we can easily determine the minimum cost of a set to be aligned if it includes only length‐1 sequences; moreover, we also can construct an optimal alignment in the most trivial way using any metric. We have also seen that for length‐2 sequences, the trivial alignment is optimal if the unit metric is used but it is not optimal for arbitrary metric. Besides that, it is also known that the trivial alignment is not always optimal for length‐3 sequences even using unit metric.

As in Example $\left(i\right)$ at the end of the Introduction, let S be as follows:

$$S=\left\{CCG,GCG,CGC\right\}.$$

Using the unit metric, the cost of the trivial alignment is 6, but it is not optimal: as we have seen, there is a non‐trivial alignment A of S so that cost $\left(A\right)$ is only 5 (see Table 7).

**TABLE 7:** The trivial and an optimal alignment of S

C	C	G	C	C	G	–
G	C	G	G	C	G	–
C	G	C	–	C	G	C

## 5 CONCLUSIONS

In this work, it was shown that the MSA problem is “easy” for length‐1 sequences and also for length‐2 sequences in special cases. While the MSA problem is well‐examined for a small number of long sequences, it is a pioneering work covering the specialties of a large number of very short sequences.

Since we know that the general problem is NP‐hard,^4^ it is still an interesting question that for how long sequences the MSA problem starts to become to be difficult? It is another open problem that in the case of length‐2 sequences, how can those metrics be characterized for which trivial alignment is always optimal for arbitrary alphabet?

## CONFLICT OF INTEREST

The authors declare no conflict of interest.

## AUTHOR CONTRIBUTIONS

KT proved the theorems, wrote the first version of this manuscript, and prepared figures. VG initiated the study, finalized the manuscript, and secured funding.
